# Structure-Based Site of Metabolism (SOM) Prediction of Ligand for CYP3A4 Enzyme: Comparison of Glide XP and Induced Fit Docking (IFD)

**DOI:** 10.3390/molecules25071622

**Published:** 2020-04-01

**Authors:** Deepak K. Lokwani, Aniket P. Sarkate, Kshipra S. Karnik, Anna Pratima G. Nikalje, Julio A. Seijas

**Affiliations:** 1 Department of Pharmaceutical Chemistry, R.C. Patel Institute of Pharmaceutical Education and Research, Shirpur, Dist-Dhule 425405, Maharashtra, India; 2 Department of Chemical Technology, Dr. Babasaheb Ambedkar Marathwada University, Aurangabad, 431004 Maharashtra, India; aniketpharma1@gmail.com (A.P.S.); karnikkshipra@yahoo.co.in (K.S.K.); 3 Wilson College, Chowpatty Seaface Road, Mumbai 400007, Maharashtra, India; annapratimanikalje@gmail.com; 4 Departamento de Química Orgánica, Facultad de Ciencias, Universidad of Santiago De Compostela, Alfonso X el Sabio, Lugo 27002, Spain; julioa.seijas@usc.es

**Keywords:** CYP3A4, Glide XP, Induced Fit Docking (IFD), Site of Metabolism (SOM)

## Abstract

Metabolism is one of the prime reasons where most of drugs fail to accomplish their clinical trials. The enzyme CYP3A4, which belongs to the superfamily of cytochrome P450 enzymes (CYP), helps in the metabolism of a large number of drugs in the body. The enzyme CYP3A4 catalyzes oxidative chemical processes and shows a very broad range of ligand specificity. The understanding of the compound’s structure where oxidation would take place is crucial for the successful modification of molecules to avoid unwanted metabolism and to increase its bioavailability. For this reason, it is required to know the site of metabolism (SOM) of the compounds, where compounds undergo enzymatic oxidation. It can be identified by predicting the accessibility of the substrate’s atom toward oxygenated Fe atom of heme in a CYP protein. The CYP3A4 enzyme is highly flexible and can take significantly different conformations depending on the ligand with which it is being bound. To predict the accessibility of substrate atoms to the heme iron, conventional protein-rigid docking methods failed due to the high flexibility of the CYP3A4 protein. Herein, we demonstrated and compared the ability of the Glide extra precision (XP) and Induced Fit docking (IFD) tool of Schrodinger software suite to reproduce the binding mode of co-crystallized ligands into six X-ray crystallographic structures. We extend our studies toward the prediction of SOM for compounds whose experimental SOM is reported but the ligand-enzyme complex crystal structure is not available in the Protein Data Bank (PDB). The quality and accuracy of Glide XP and IFD was determined by calculating RMSD of docked ligands over the corresponding co-crystallized bound ligand and by measuring the distance between the SOM of the ligand and Fe atom of heme. It was observed that IFD reproduces the exact binding mode of available co-crystallized structures and correctly predicted the SOM of experimentally reported compounds. Our approach using IFD with multiple conformer structures of CYP3A4 will be one of the effective methods for SOM prediction.

## 1. Introduction

Cytochrome P450 3A4 (CYP3A4) is the most important enzyme in the superfamily Cytochrome P450, besides endogenous compounds, that metabolizes about half of the currently marketed drugs [1]. The inhibition of biotransformation mediated by CYP3A4 is known to be a significant mechanism for undesirable side effects of drugs since this enzyme is responsible for the metabolism of 50%–60% of oral drugs on the market, including anesthetics, antibiotics, steroids, and cancer chemotherapeutics [2]. For a given substrate, cytochrome P450 (CYP) enzymes can catalyze a wide variety of reactions, such as hydroxylation, epoxidation or heteroatom oxidation, dealkylations, and desaturation [3]. The CYPs metabolize foreign compounds into polar hydrophilic metabolites by integrating one oxygen atom into the substrate, which makes it water-soluble and, consequently, more easily excreted [4,5]. The CYP3A4 exhibits very wide ligand specificity and catalyzes a large number of chemical processes. Therefore, inhibition of the metabolic activity of CYP3A4 by one substrate can extensively influence the metabolism of other substrates. For this reason, the prediction of CYP inhibition by compounds and/or its metabolic stability along with other ADMET profiles are important for assessing the quality of the lead molecule. To reduce the CYP inhibition and enhance the metabolic stability of compounds, the prediction of sites of metabolism (SOM) or binding modes with CYPs are very helpful. Using the predicted SOM, the substitution around the molecule sites can be converted into a more stable metabolically functional group. The CYP3A4 substrates are an extremely varied group of molecules, even though they are typically polar, aromatic, and have a higher molecular weight than most other CYP substrates [1]. In order to accommodate such large and diverse molecules, the protein must undergo conformational changes, as suggested by Williams et al. [6]. Thus, in order to design new compounds, which are less prone to metabolism, it is necessary to know SOM in their structure. The SOM can be identified by predicting the accessibility of the substrate’s atom toward the oxygenated Fe atom of heme in a CYP protein.

**Figure 1:**
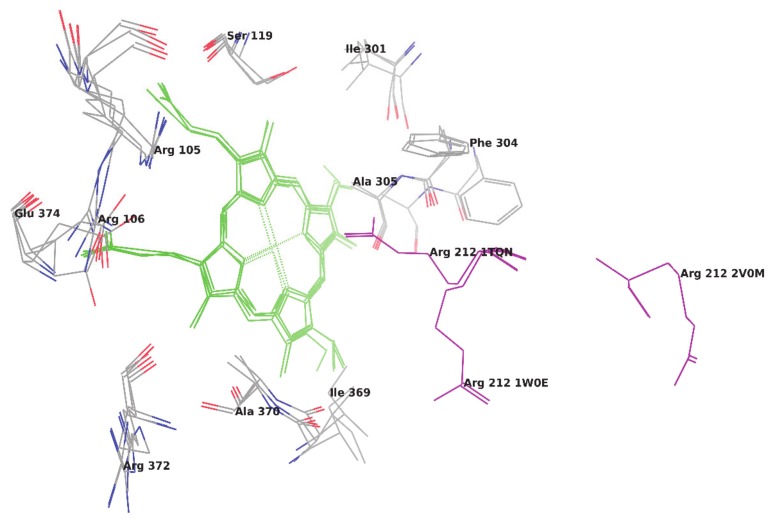
Superimposition of three crystal structures of CYP3A4.

To metabolize such a variety of compounds, CYP3A4 has to be an extremely flexible structure and its X-ray crystallographic structures have shown that the CYP3A4 can adopt substantially different conformations depending on the ligand with which it is being co-crystallized [6]. The available native (ligand unbound) and ligand-bound crystal structures of CYP3A4 in Protein Data Bank (PDB) have shown the high flexibility of amino acid residues in the side chain and loop region, which makes a binding pocket in a closed and open conformation, respectively. As reported by Yuki H. et al. [7], we also superimposed six different crystal structures of CYP3A4 and root-mean-square deviation (RMSD) between the docked ligand poses and their native poses were calculated. The RMSD values of the superimposition of C-alpha carbon atoms of crystal structures were obtained in a range of 1.58 Å to 10.54 Å (Table 1). Based on RMSD values, it is cleared that the CYP enzyme is highly flexible and confirmed that every ligand induces different conformational changes into the binding pocket of the CYP3A4 enzyme (Figure 1) and, thus, make ligand-enzyme interaction studies difficult. The main difference found between two unbound crystal structures of CYP3A4 is in the orientation of Arg212. In one ligand unbound structure (PDB code 1W0E), Arg 212 is orientated away from the heme group and, in another structure (Pdb code 1TQN), it occupies the orientation toward the heme group [8]. Whereas, in a structure bounded with ketoconazole (PDB code 2V0M), CYP3A4 has many conformational changes and Arg212 is located away from the active site. Some hydrophobic cluster in the above ketoconazole bound structure is shown broken, which increases the volume of the active site. Dan Fishelovitch et al. have reported that Arg212 may strongly interact with Phe304 and, thus, affect the binding of substrates/inhibitors on the enzyme [8].

The number of approaches has been investigated to predict SOM in the ligand and to visualize the accessibility of SOM near to heme region [7,9,10,11,12]. Yuki H. et al. carried out Molecular Dynamic (MD) simulations initiated from multiple CYP3A4-carbamazepine complexes to predict the approachability of carbamazepine atoms near the heme Fe [7]. The above approach correctly predicted the SOM in carbamazepine, which is experimentally oxidized by CYP3A4 but performing MD simulation using multiple initial structures is the time-consuming calculations. Rydberg P. et al. developed a ligand-based method based on the SMARTCyp approach. The above method predicts the SOM in ligand oxidized by cytochrome P450 2D6 directly from the 2D structure of a molecule using only two descriptors, which include the distance to a protonated nitrogen atom and the distance to the end of the molecule [10].

In this study, we have taken six PDBs of CYP3A4 and first performed re-docking of all bound co-crystallized ligands for validation of docking methodology as well as for regeneration of the bound pose of the ligand. Afterward, the cross-docking studies were performed using co-crystallized ligands over all six PDBs using Glide XP and Induced fit docking methodologies. For re-docking and cross-docking studies, RMSD between co-crystallized and docked ligand was calculated and considered as the parameter for examining the accuracy of docking studies. Lastly, studies were extended for the prediction of SOM for 10 different ligands obtained from literature whose experimental SOM is reported. The overall study involves the comparison of both Glide extra precision (XP) and Induced fit docking (IFD) methodologies for the correct prediction of SOM. As per the literature [11], the docking methodology was considered successful if the distance between SOM of the ligand and the Fe atom of heme is within 6.0 Å, but we did not set any limit and considered that SOM must be closer to heme for the metabolism of any compound. 

## 2. Materials and Methods

### 2.1. Glide Ligand Docking

Molecular docking studies were performed in Maestro 9.1 using Glide v6.8 (Schrodinger, LLC, New York, NY, USA). All compounds were built using Maestro build panel and optimized to lower energy conformers using Ligprep v3.5.9 (Schrodinger, LLC). The PDB’s 1W0F (Progesterone), 1W0G (Metyrapone), 2V0M (Ketoconazole), and 3NXU (Ritonavir) were taken from RCSB Protein Data Bank and prepared for docking using ‘protein preparation wizard’ in Maestro v10.3. (Schrodinger, LLC). The bond orders and formal charges were added for hetero groups and hydrogens were added to all atoms in the structure. The side chains that are not close to the binding cavity and do not participate in salt bridges were neutralized and termini were capped by adding N-acetyl (ACE) and N-methyl amide (NMA) residue. After preparation, the structure was refined to optimize the hydrogen bond network using the OPLS_2005 force field. The minimization was terminated when the energy converged or the RMSD reached a maximum cutoff of 0.30 Å. The extra precision (XP) docking mode for all compounds was performed on the generated grid of the protein structure. The final evaluation of ligand-protein binding was done with the Glide score.

### 2.2. Induced Fit Docking

IFD was performed using the module Induced Fit Docking of Maestro v9.1. (Schrodinger, LLC). The entire receptor molecule constrained and minimized with an RMSD cutoff of 0.18 Å, which was selected for generation of the centroid of the residues and the box size was generated automatically. The initial Glide docking for each ligand was carried out. The side chains were trimmed automatically based on the B-factor, with receptor and ligand van der Waals scaling from 0.70 to 0.50, respectively. The number of poses generated was set to be 20. The prime side-chain prediction and minimization were carried out in which residues were refined within 5.0 Å of ligand poses and side chains were optimized. This leads to a ligand structure and conformation that is induced to fit to each pose of the receptor structure. Lastly, Glide XP redocking was carried out into structures within 30.0 kcal/mol of the best structure, and within the top 20 structures overall. The ligand was rigorously docked into the induced-fit receptor structure and the results yielded an IFD score for each output pose.

## 3. Result and Discussion

### 3.1. Re-Docking Study

The re-docking study can replicate co-crystallized binding geometry and an associated ligand’s orientation state. It also provides the easiest possible case, where the ligand and protein are presented in their right, binding conformations. This study supports by avoiding the omnipresent need to change at least the side-chain conformations of the ligand and protein to form the correct complex. This study also helps to appropriately score the correct complex for most scoring functions than to classify a complex that has one or more functional groups mistaken, as is usual for docking.

Therefore, the re-docking study was conducted to review the docking methodology to predict the correct ligand pose within the active enzyme site. Out of four ligand-bound crystal structures, the structure belonging to the PDB ID 1W0F was not chosen for re-docking since the inbound ligand Progesterone is present at the peripheral site, which is 17 Å away from the heme. For the remaining three ligand-bound crystal structures, the inbound ligand was extracted and re-docked using Glide XP and induced fit docking (IFD) technique. The number of poses for each ligand were generated by both methodologies. For each docked pose, the RMSD was calculated by superimposing on their respective inbound crystallized ligand. The best pose of ketoconazole, metyrapone, and ritonavir was confirmed based on their lowest RMSD value when superimposed on their respective inbound crystallized ligand (Table 2). The lowest RMSD of superimposition was found to be 2.12 Å, 2.55 Å, and 2.31 Å by Glide XP for ketoconazole, metyrapone, and ritonavir, respectively. However, IFD reproduces the poses of ketoconazole, metyrapone, and ritonavir with RMSD of 1.18 Å, 2.23 Å, and 2.39 Å, respectively. To compare the docking methodology, the mean RMSD was also calculated for ligands after averaging the RMSD obtained by the superimposition of all poses to the respective co-crystallized bound ligand. After visualization of docking poses as well as the assessment of the mean and lowest RMSD value of all compounds, it was observed that IFD is more thoroughly predicted and recollected the accurate poses of the ligand. Although the mean RMSD obtained by the superimposition of poses of Ritonavir over its co-crystallized structure in PDB 3NXU was higher for IFD as compared to Glide XP docking, both methodologies predict the accurate poses for Ritonavir.

### 3.2. Cross-Docking Studies

Cross-docking is intended to classify putative ligands for each target being examined. The multiple receptor conformation approaches of the same enzyme in a cross-docking study validated the normal docking technique. The performance of the cross-docking methodology will be established by estimating the RMSD of the docked pose of the ligand in all protein conformers concerning its reported crystal conformer. RMSD values below 2.0 Å are considered effective, but values that are closest to zero are optimal.

All three ligands including ketoconazole, metyrapone, and ritonavir have been docked to all six crystal structures to further validate both the IFD and Glide XP docking procedures. Similar to re-docking studies, the RMSD value was determined by superimposing docked poses of each compound from all ligand-enzyme conformers complex over their respective bound ligand pose. Table 3 outlines the RMSD values of docked poses of each compound in all six crystal structures (PDBs) when superimposed on their respective bound ligand pose. In most PDBs, IFD regenerates the correct poses for ketoconazole and metyrapone with an RMSD value for best pose below 2.0 Å whereas the best pose predicted by Glide XP has an RMSD value above 2.0 Å. However, for ritonavir, both IFD and Glide XP fail to regenerate the correct pose except for PDB Id 3NXU. 

**Figure 2:**
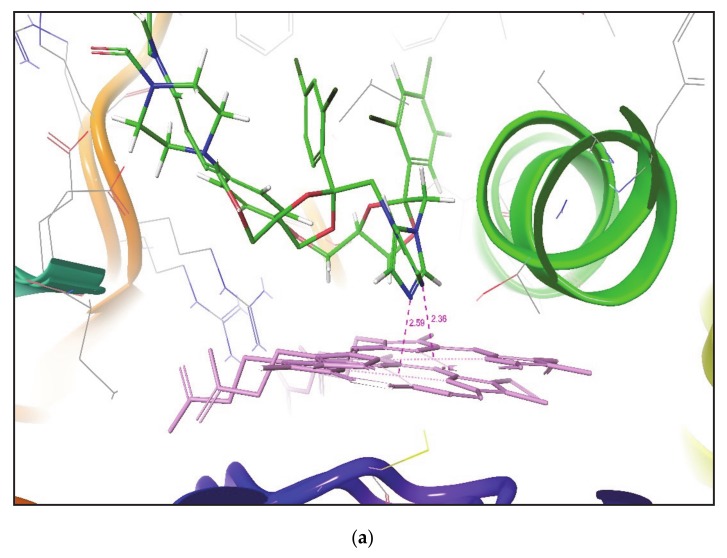

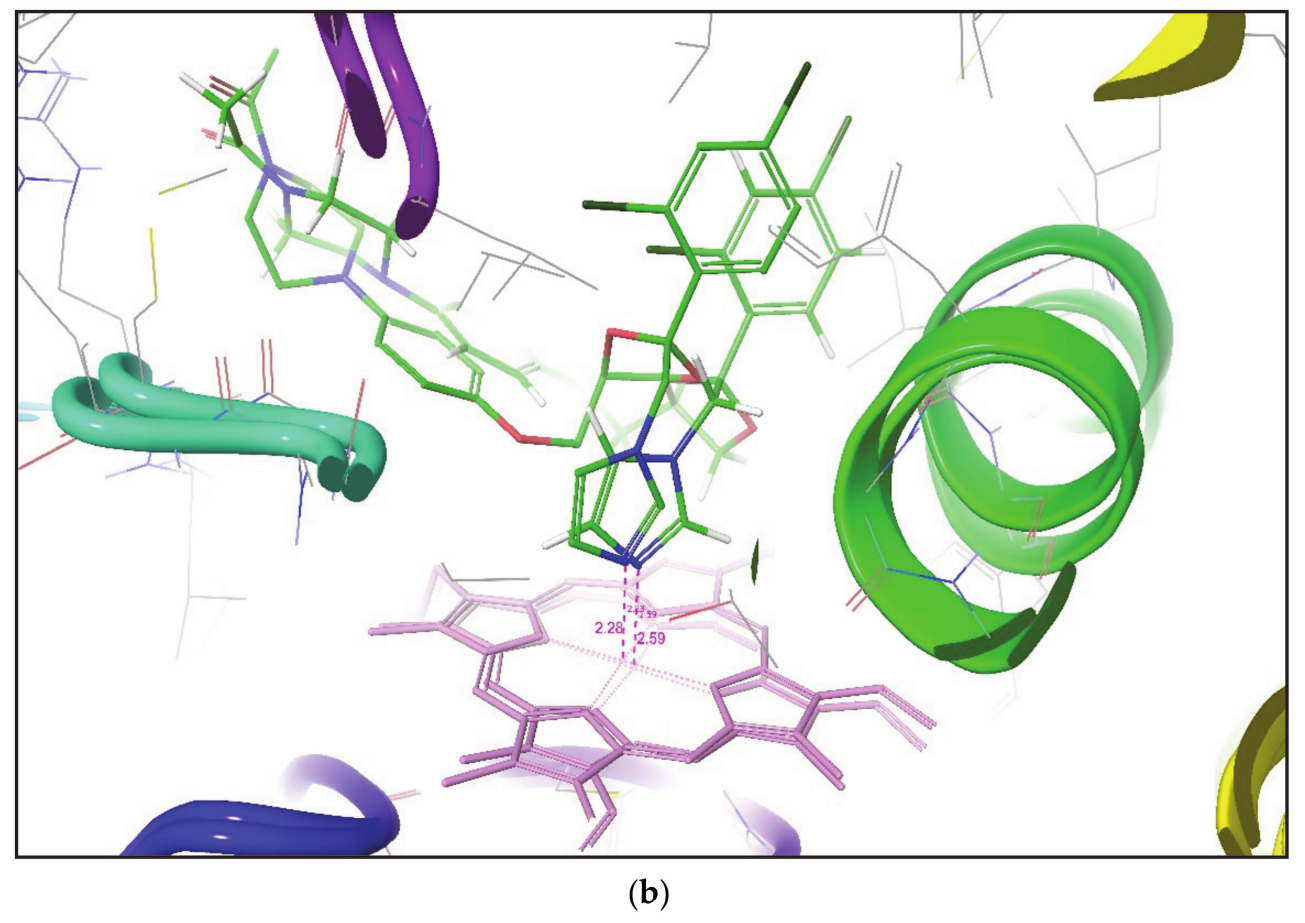
Superimposition of the pose of ketoconazole docked in (**a**) PDB id 1TQN and (**b**) PDB id 2V0M by IFD over crystal structure of ketoconazole bound CYP3A4 (PDB id 2V0M). The pink dotted line indicates the distance between site of metabolism (SOM) and the Fe atom of heme.

By observing the lowest RMSD and mean RMSD value for docked poses of ketoconazole and metyrapone from cross-docking results, it can be noted that IFD identifies similar binding poses of both compounds in all protein conformers, as reported in their corresponding crystal structures. Figure 2 shows the superimposition of the docked pose of ketoconazole over their corresponding crystal structures. It was again noticed that the IFD gives a similar pose of the compound in both closed and open conformation of the CYP3A4 crystal structure.

### 3.3. Prediction of Site of Metabolism (SOM)

The SOM refers to the place where the metabolic reaction takes place in a molecule. The recognition of CYP-mediated SOM is generally a starting point in the investigation of the metabolic pathway and may aid in the optimization of the lead molecule. The active metabolite can be used as a lead molecule if it has enhanced pharmacological, pharmacokinetic, and toxicological profiles compared to the parent. However, if a metabolite is unwanted, the SOM information may further direct the modification of the structure in a direction to eliminate or replace unstable sites to avoid such bio-transformations [13]. The metabolism is the process of breaking old chemical bonds and forming new bonds in the presence of an enzyme. Therefore, within a molecule, it is faithful to identify the chemical bond and/or atom or group of atoms where the metabolic reaction occurred as the metabolism site. The Information of SOM always plays an important role in studying the metabolic mechanism of xenobiotics and provides valuable information to design and refine products rationally [9].

**Figure 3:**
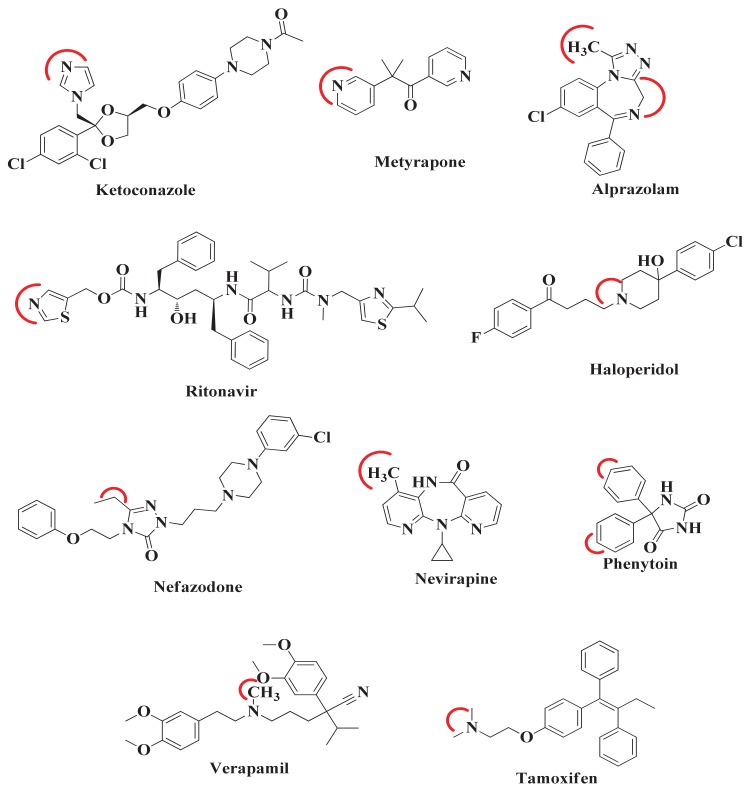
Chemical Structures of compounds. Red color indicates experimentally reported major SOM.

After the comparison and validation of both docking methodologies, we extend our aim to predict the desired metabolic pose and SOM in those compounds whose ligand-enzyme complex crystal structures are not available in PDB. For this, the 10 compounds were selected from literature. The SOM of these compounds was reported by experimental methodology [14,15,16,17,18,19], but their bound co-crystallized CYP3A4 structure is not available except for ketoconazole, metyrapone, and ritonavir (Figure 3). The red curved line around each compound indicates the SOM. Therefore, when these compounds will be docked to CYP3A4, it would be expected that these sites would come close to and interact with the heme atom Fe. 

In order to predict the desired metabolic pose where SOM of each compound positioned near the heme, all 10 compounds were docked in all six CYP3A4 crystal structures by both induced fit and Glide XP methodologies. For each compound, the best ligand-enzyme complexes in each conformer (crystal structure) were selected according to the desired metabolic pose of the drug candidate and further analyzed (Table 4). Table 4 states that, for each compound docked by IFD to different CYP3A4 conformers, the identification of desired metabolic poses was much greater in number than that found by Glide XP docking. The distance between SOM and the Fe atom of heme was also measured. It was noted that, in all PDBs, IFD obtained the optimal metabolic poses for Ketoconazole with a distance between SOM and the Fe atom of heme in between 2.15 to 2.40 Å. Whereas Glide XP generates a few desired poses for Ketoconazole in all PDBs with a minimum distance of 2.52 Å between SOM and the Fe atom of heme in PDB ID 2V0M. Both IFD and Glide XP docking methodologies generated only a single desired metabolic pose for Metyrapone in some of the CYP3A4 conformers, but, in IFD suggested poses, SOM is found to be within 2.40 Å of the heme region. However, only a single Glide XP pose for Metyrapone in CYP3A4 Conformer (PDB ID 1W0G) is at a distance of 2.69 Å from the Fe atom of heme. In the crystal structure of CYP3A4 (PDB Id 3NXU), SOM of Ritonavir is found at a distance of 2.42 Å from the Fe atom. For Ritonavir, both IFD and Glide XP generated several metabolic poses in all CYP3A4 conformers. However, IFD generated more desired metabolic poses than Glide XP in all CYP3A4 crystal structures except for PDB Id 1W0F. It was also observed that IFD placed SOM of Ritonavir at a distance of 1.92 Å from the Fe atom in one of the CYP3A4 conformers (PDB ID 2V0M), which is found to be closer to heme than that reported in its CYP3A4 crystal structure (PDB ID 3NXU).

While comparing the above approach with all the remaining seven small molecules whose crystallographic ligand-CYP3A4 complex structures are unavailable in PDB, it was found that both IFD and Glide XP docking methodologies lack in the generation of ideal metabolic poses at a distance of 3.0 Å of SOMs from the heme Fe atom in all CYP3A4 conformational structures. Despite this, IFD effectively placed SOM of Tamoxifen in front of heme Fe to a minimum distance range of 2.19-2.38 Å in all CYP3A4 conformational structures. Whereas Glide XP produced only one Tamoxifen pose in each CYP3A4 conformer and put SOM of Tamoxifen near the heme at a distance of 2.96 Å in one of CYP3A4 (PDB ID 1W0E) conformational structures. For Alprazolam and Haloperidol, IFD generates the number of desired metabolic poses in each of CYP3A4’s conformations compared to that generated by Glide XP, but both methodologies failed to place SOM of both compounds close to the Fe atom of heme. Similarly, IFD established the number of ideal metabolic poses for Nefazodone, Nevirapine, and Phenytoin, but was unable to locate SOM at the desired distance with the heme Fe atom. Unfortunately, both IFD and Glide XP in each CYP3A4 conformer produced the number of poses for Verapamil but were unable to identify a single desired metabolic pose.

**Figure 4:**
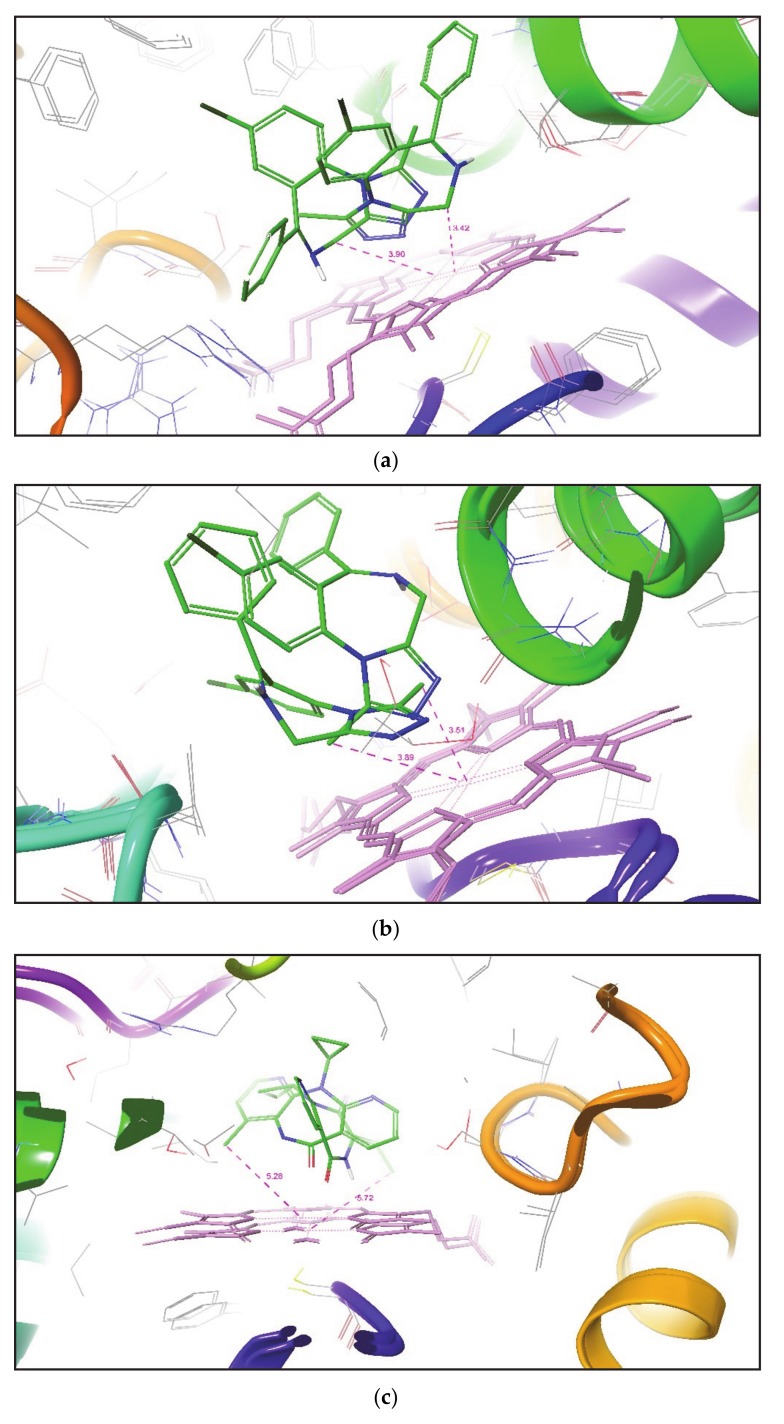

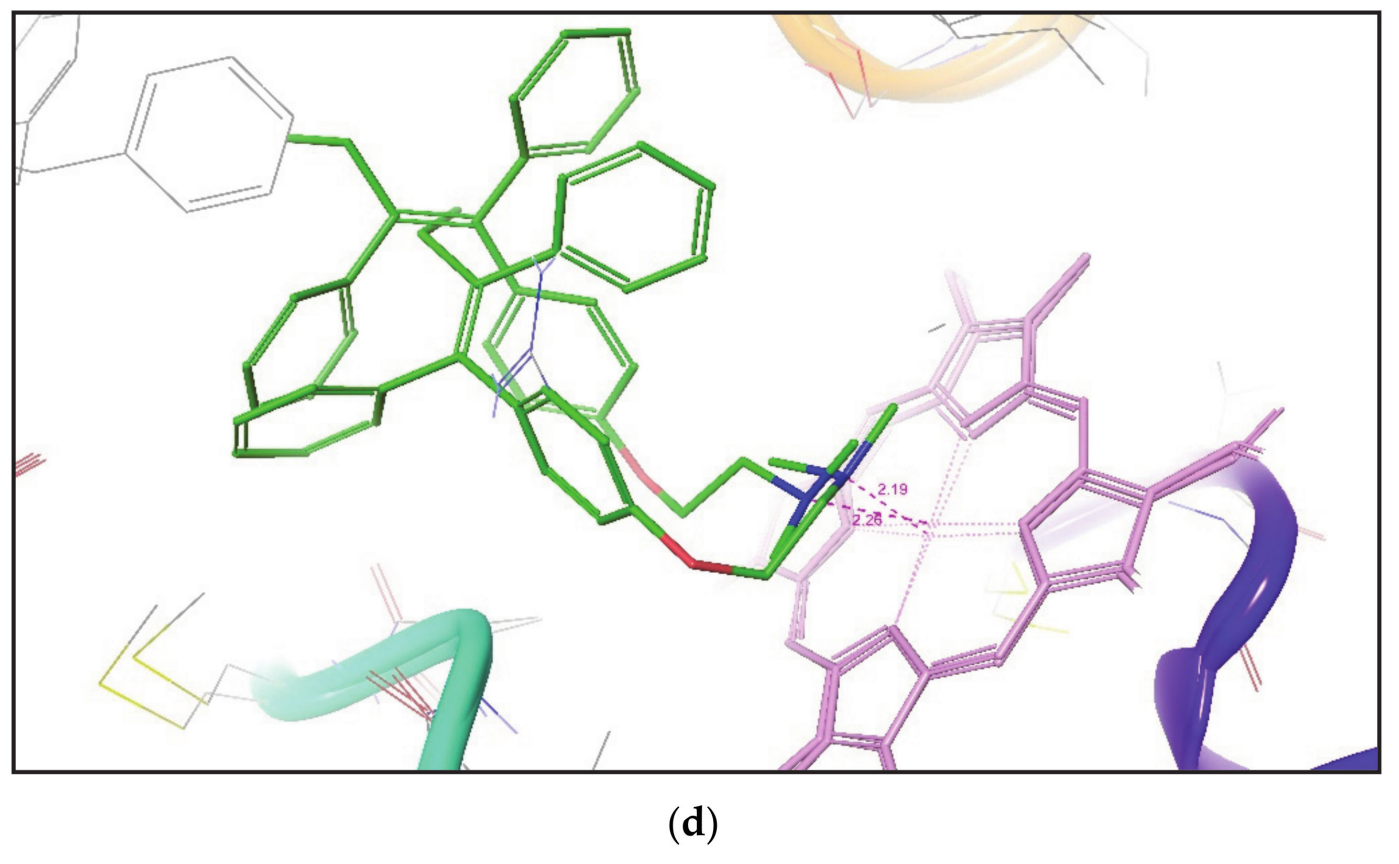
Superimposition of the IFD docking pose of ligands in CYP3A4 enzyme (**a**) Alprazolam (1W0E-1W0G)-Site 1, (**b**) Alprazolam (1W0E-1W0G)-Site 2, (**c**) Nevirapine (1W0F-3NXU), and (**d**) Tamoxifen (1W0G-1TQN). The pink dotted line indicates the distance between SOM and the heme Fe atom.

Figure 4. This figure outlines the superimposition of Alprazolam, Nevirapine, and Tamoxifen in the active site of different CYP3A4 conformation. After analyzing the overall results, both methodologies do not place SOM of some compounds within a distance of 2.5 Å to the heme Fe atom, but it has been found that induced fit docking (IFD) is better than Glide XP to produce favorable metabolic poses for most of the compounds in the active site of CYP3A4.

Similar to Yuki H. et al. [7], Teixeira V.H. et al. also employed multi-conformational docking where all conformations are generated by several molecular dynamic simulations to analyze the binding modes of various ligands, and noted that the docking is considered successful if the SOM is found within 6.0 Å of the heme Fe [11]. By comparing with the above approach, it can also be revealed that a less time-consuming IFD approach also placed the SOM of all ligands in all CYP3A4 conformers within a distance of 6.0 Å from the Fe atom of heme.

Tarcsay A. et al. developed a structure-based approach for predicting SOM involving the use of MetabolExpert for the generation of metabolite and Glide for docking of substrate and predicted metabolites to the CYP binding site. They applied the distance filter of 6.0 Å and concluded that the metabolite docking approach is significantly more accurate than substrate docking with an accuracy rate of 84%. If comparing the above approach with IFD, it can be revealed that IFD can produce desired metabolic poses and predicted SOM correctly for nine out of ten compounds with an accuracy rate of 90%. Furthermore, the use of IFD instead of Glide docking does not necessarily require us to generate metabolites. Direct IFD docking of a substrate can also predict SOM.

## 4. Conclusions

The present work illustrates docking of 10 ligands on six different conformers of CYP3A4 and the prediction of the desired metabolic pose and SOM of the ligands. The Glide XP and Induced Fit docking (IFD) tool of Schrodinger software suite was employed to predict the binding mode of all ligands into six X-ray crystallographic structures of CYCP3A4. Since CYP3A4 is an extremely flexible enzyme, its active site cavity shows significant changes in volume and can be exchanged between large numbers of different conformations. It has been found that docking ligands in different conformers are highly advantageous, as some of them may have high affinities for particular conformers, which helps predict the desired metabolic pose. In each docked complex, the distance between the heme Fe atom of CYP3A4 and the SOM of ligands was measured. Since the protein flexibility issue is concerned with CYP3A4, the Glide XP docking was unable to predict the desired metabolic pose of some of the ligands and IFD produced at least one desired ligand-enzyme complex for all ligands. As a result, IFD was found to be one of the reliable methods for predicting and analyzing SOMs of the ligand in the flexible binding pocket of CYP3A4. The IFD methodology can, therefore, be applied to new compounds for predicting the desired metabolic pose and SOM.

## Figures and Tables

**Table 1:** Superimposition of C-alpha carbon atoms of the crystal structure of CYP3A4.

	1TQN ^1^	1W0E ^1^	1W0F ^2,3^	1W0G ^2^	2V0M ^2^	3NXU ^2^
**1TQN**	-	7.23	7.25	7.40	6.16	4.58
**1W0E**	7.23	-	1.66	2.10	10.54	8.65
**1W0F**	7.25	1.66	-	1.58	10.59	8.72
**1W0G**	7.40	2.10	1.58	-	10.67	8.83
**2V0M**	6.16	10.54	10.59	10.67	-	4.19
**3NXU**	4.58	8.65	8.72	8.83	4.19	-

1. Native (ligand unbound) Crystal Structures. PDB ids 1TQN and 1W0E. 2. Ligand bound Crystal Structures: PDB ids 1W0F (Progesterone), 1W0G (Metyrapone), 2V0M (Ketoconazole), and 3NXU (Ritonavir). 3. PDB Id 1W0F: Progesterone bound at a peripheral site, 17 Å away from heme.

**Table 2:** Superimposition of ligands after docking over the corresponding crystal structure of CYP3A4.

PDB ID	RMSD After Superimposition on Crystal Structure (Å)					
	IFD			Glide XP		
	Max.	Min.	Mean	Max.	Min.	Mean
**Ketoconazole**						
2V0M	3.34	1.18	2.11	2.17	2.12	2.14
**Metyrapone**						
1W0G	2.23	2.23	2.23	2.55	2.55	2.55
**Ritonavir**						
3NXU	6.21	2.39	4.36	5.36	2.31	3.79

**Table 3:** Superimposition of ligands after docking the overall crystal structure of CYP3A4.

PDB ID	RMSD after Superimposition on the Crystal Structure (Å)					
	IFD			Glide XP		
	Max.	Min.	Mean	Max.	Min.	Mean
**Ketoconazole**						
1TQN	3.41	1.98	2.60	3.52	2.65	3.08
1W0E	3.12	1.76	2.38	2.79	2.79	2.79
1W0F	3.64	2.30	3.02	3.49	3.10	3.25
1W0G	4.06	1.82	2.87	2.83	2.83	2.83
2V0M	3.34	1.18	2.11	2.17	2.12	2.14
3NXU	3.78	2.26	3.11	3.59	3.24	3.42
**Metyrapone**						
1TQN	-	-	-	-	-	-
1W0E	1.89	1.89	1.89	-	-	-
1W0F	-	-	-	2.30	2.30	2.30
1W0G	2.23	2.23	2.23	2.55	2.55	2.55
2V0M	1.39	1.39	1.39	2.51	2.51	2.51
3NXU	2.18	2.18	2.18	-	-	-
**Ritonavir**						
1TQN	4.92	4.33	4.67	4.27	4.27	4.27
1W0E	4.61	3.49	4.05	4.69	3.91	4.15
1W0F	-	-	-	4.21	4.00	4.10
1W0G	4.99	3.88	4.43	4.59	3.74	4.09
2V0M	5.62	3.31	4.32	5.01	2.98	3.89
3NXU	6.21	2.39	4.36	5.36	2.31	3.79

**Table 4:** Comparison of IFD and Glide XP results for docking of ligands in the active site of CYP3A4.

PDB ID	IFD					Glide XP				
	Generated Pose	Desired Metabolic Pose	Distance of Atom from Heme Fe (Å)			Generated Pose	Desired Metabolic Pose	Distance of Atom from Heme Fe (Å)		
			Max	Min	Mean			Max	Min	Mean
**Ketoconazole (PDB Code 2V0M, Dist from Heme Fe: 2.59Å)**										
1TQN	21	5	2.40	2.17	2.32	8	2	3.78	3.21	3.49
1W0E	27	11	2.39	2.21	2.33	6	1	3.26	3.26	3.26
1W0F	31	6	2.40	2.33	2.36	8	3	3.92	3.61	3.76
1W0G	42	15	2.40	2.19	2.32	4	1	3.82	3.82	3.82
2V0M	88	18	2.39	2.15	2.31	3	2	2.81	2.52	2.66
3NXU	88	16	2.40	2.28	2.36	4	4	3.92	3.40	3.56
**Metyrapone (PDB Code 1W0G, Dist from Heme Fe: 2.36Å)**										
1TQN	15	0	-	-	-	2	0	-	-	-
1W0E	9	1	2.34	2.34	2.34	0	0	-	-	-
1W0F	17	0	-	-	-	2	1	3.54	3.54	3.54
1W0G	3	1	2.39	2.39	2.39	1	1	2.69	2.69	2.69
2V0M	17	1	2.39	2.39	2.39	1	1	3.35	3.35	3.35
3NXU	22	1	2.39	2.39	2.39	1	0	-	-	-
**Ritonavir (PDB Code 3NXU, Dist from Heme Fe: 2.42Å)**										
1TQN	4	3	2.34	2.09	2.24	32	1	3.20	3.20	3.20
1W0E	4	2	2.27	2.17	2.22	10	4	3.68	2.83	3.34
1W0F	0	0	-	-	-	32	3	3.57	3.23	3.35
1W0G	23	2	2.37	2.35	2.36	14	7	3.64	2.53	3.00
2V0M	58	26	2.40	1.92	2.31	29	18	3.73	2.47	3.00
3NXU	61	53	2.40	1.96	2.30	24	12	3.99	2.65	3.21
**Alprazolam (includes both SOM)**										
1TQN	6	6	4.31	3.90	4.14	2	0	-	-	-
1W0E	52	52	4.77	3.42	4.18	2	2	3.90	3.84	3.87
1W0F	28	28	4.36	3.81	3.99	2	1	4.66	4.66	4.66
1W0G	72	72	4.52	3.55	4.07	1	1	4.58	4.58	4.58
2V0M	78	78	4.66	3.30	4.03	1	0	-	-	-
3NXU	74	74	4.65	3.28	4.15	1	1	3.56	3.56	3.56
**Haloperidol**										
1TQN	40	2	5.33	5.33	5.33	2	0	-	-	-
1W0E	82	38	5.98	5.45	5.75	0	0	-	-	-
1W0F	78	12	5.99	5.66	5.91	3	0	-	-	-
1W0G	72	10	5.85	5.37	5.68	0	0	-	-	-
2V0M	96	26	5.99	4.91	5.63	1	0	-	-	-
3NXU	52	10	5.75	5.49	5.64	3	2	5.73	5.64	5.68
**Nefazodone**										
1TQN	2	0	-	-	-	2	0	-	-	-
1W0E	8	0	-	-	-	0	0	-	-	-
1W0F	24	2	5.25	5.25	5.25	2	2	6.00	6.00	6.00
1W0G	60	6	5.74	5.12	5.44	1	0	-	-	-
2V0M	12	4	6.00	5.11	5.55	1	1	3.98	3.98	3.98
3NXU	32	32	4.15	3.32	3.72	0	0	-	-	-
**Nevirapine**										
1TQN	9	0	-	-	-	2	0	-	-	-
1W0E	26	6	5.59	5.42	5.51	0	0	-	-	-
1W0F	40	14	5.96	5.28	5.62	2	0	-	-	-
1W0G	62	30	5.95	4.76	5.40	0	0	-	-	-
2V0M	74	30	5.94	4.98	5.56	0	0	-	-	-
3NXU	16	4	5.95	5.72	5.83	0	0	-	-	-
**Phenytoin**										
1TQN	1	0	-	-	-	3	0	-	-	-
1W0E	62	0	-	-	-	1	0	-	-	-
1W0F	52	0	-	-	-	3	2	4.50	3.62	4.06
1W0G	60	0	-	-	-	2	0	-	-	-
2V0M	84	16	5.91	5.70	5.84	2	0	-	-	-
3NXU	80	6	5.78	5.16	5.57	3	0	-	-	-
**Tamoxifen**										
1TQN	10	10	2..37	2.26	2.32	2	0	-	-	-
1W0E	2	2	2.33	2.33	2.33	1	1	2.96	2.96	2.96
1W0F	38	34	5.00	2.19	2.79	2	0	-	-	-
1W0G	60	46	4.96	2.19	4.06	0	0	-	-	-
2V0M	18	18	4.61	2.38	2.88	1	1	4.26	4.26	4.26
3NXU	50	32	2.40	2.35	2.38	2	1	3.85	3.85	3.85
**Verapamil**										
1TQN	46	0	-	-	-	6	0	-	-	-
1W0E	80	0	-	-	-	6	0	-	-	-
1W0F	48	0	-	-	-	5	0	-	-	-
1W0G	72	0	-	-	-	6	0	-	-	-
2V0M	146	0	-	-	-	6	0	-	-	-
3NXU	128	0	-	-	-	6	0	-	-	-
